# Valproic acid attenuates traumatic spinal cord injury-induced inflammation via STAT1 and NF-κB pathway dependent of HDAC3

**DOI:** 10.1186/s12974-018-1193-6

**Published:** 2018-05-18

**Authors:** Shoubo Chen, Jingfang Ye, Xiangrong Chen, Jinnan Shi, Wenhua Wu, Wenping Lin, Weibin Lin, Yasong Li, Huangde Fu, Shun Li

**Affiliations:** 1 Department of Orthopaedics, The Second Affiliated Hospital, Fujian Medical Universityz, Quanzhou, 362000 Fujian Province China; 2 Department of nursing faculty, Quanzhou Medical College, Quanzhou, 362000 Fujian Province China; 3 Department of Neurosurgery, The Second Affiliated Hospital, Fujian Medical University, Quanzhou, 362000 Fujian Province China; 4 Department of Neurosurgery, Affiliated Hospital of YouJiang Medical University for Nationalities, Baise, 533000 Guangxi Province China; 5 Department of Neurosurgery, Affiliated Hospital of North Sichuan Medical College, Nanchong, 637000 Sichuan Province China

**Keywords:** Spinal cord injury, Valproic acid, HDAC3, Microglia, Inflammatory, STAT1, NF-κB pathway

## Abstract

**Background:**

Microglial polarization with M1/M2 phenotype shifts and the subsequent neuroinflammatory responses are vital contributing factors for spinal cord injury (SCI)-induced secondary injury. Nuclear factor-κB (NF-κB) is considered the central transcription factor of inflammatory mediators, which plays a crucial role in microglial activation. Lysine acetylation of STAT1 seems necessary for NF-kB pathway activity, as it is regulated by histone deacetylases (HDACs). There have been no studies that have explained if HDAC inhibition by valproic acid (VPA) affects the NF-κB pathway via acetylation of STAT1 dependent of HDAC activity in the microglia-mediated central inflammation following SCI. We investigated the potential molecular mechanisms that focus on the phenotypic transition of microglia and the STAT1-mediated NF-κB acetylation after a VPA treatment.

**Methods:**

The Basso-Beattie-Bresnahan locomotion scale, the inclined plane test, the blood-spinal cord barrier, and Nissl staining were employed to determine the neuroprotective effects of VPA treatment after SCI. Assessment of microglia polarization and pro-inflammatory markers, such as tumor necrosis factor (TNF)-α, interleukin (IL)-1β, IL-6, and interferon (INF)-γ was used to evaluate the neuroinflammatory responses and the anti-inflammatory effects of VPA treatment. Immunofluorescent staining and Western blot analysis were used to detect HDAC3 nuclear translocation, activity, and NF-κB signaling pathway activation to evaluate the effects of VPA treatment. The impact of STAT1 acetylation on NF-kB pathway and the interaction between STAT1 and NF-kB were assessed to evaluate anti-inflammation effects of VPA treatment and also whether these effects were dependent on a STAT1/NF-κB pathway to gain further insight into the mechanisms underlying the development of the neuroinflammatory response after SCI.

**Results:**

The results showed that the VPA treatment promoted the phenotypic shift of microglia from M1 to M2 phenotype and inhibited microglial activation, thus reducing the SCI-induced inflammatory factors. The VPA treatment upregulation of the acetylation of STAT1/NF-κB pathway was likely caused by the HDAC3 translocation to the nucleus and activity. These results indicated that the treatment with the VPA suppressed the expression and the activity of HDAC3 and enhanced STAT1, as well as NF-κB p65 acetylation following a SCI. The acetylation status of NF-kB p65 and the complex with NF-κB p65 and STAT1 inhibited the NF-kB p65 transcriptional activity and attenuated the microglia-mediated central inflammatory response following SCI.

**Conclusions:**

These results suggested that the VPA treatment attenuated the inflammatory response by modulating microglia polarization through STAT1-mediated acetylation of the NF-κB pathway, dependent of HDAC3 activity. These effects led to neuroprotective effects following SCI.

## Background

Traumatic spinal cord injury (SCI) initiates a series of cellular and molecular events, including microglial activation, inflammatory response, and abnormal mitochondrial activities, which induce neuronal death and lead to permanent neurological deficits [1–3]. SCI-induced microglial activation and subsequent release of inflammatory factors, such as interleukin (IL), tumor necrosis factor (TNF), and interferon (INF), cause direct neuronal death while inducing vascular endothelial cells to express a variety of cell adhesion and chemotaxis molecules [3–5]. These pro-inflammatory factors stimulate nitric oxide synthesis, which leads to increased capillary permeability and blood-spinal cord barrier dysfunction while promoting neuronal apoptosis [2, 3]. The inhibition of SCI-induced, the microglial activation, and the subsequent neuroinflammatory response have been shown to improve the recovery in SCI patients [6, 7].

Neuroinflammatory responses induced by activated microglia, via the NF-κB pathway, are the critical contributing factors of secondary injury [8, 9]. The NF-κB signaling pathway is passively released after an injury by necrotic or damaged cells, which activates microglia to secrete a large number of inflammatory cytokines, as well as cascade amplification of inflammatory responses [8–10]. The NF-κB exists in an inactive state sequestered in the cytoplasm by the NF-κB (IκB) inhibitor during non-inflammatory conditions. The activation of the NF-κB is initiated by IκB kinase (IKK), which degrades cytoplasmic IκB protein, which triggers the rapid release of NF-κB from IκB and intranuclear translocation [11, 12]. The activation process co-exists with signal transducers and activators of transcriptions (STATs) [13, 14]. The DNA-binding STATs enter from the cytoplasm to the nucleus and activate their target genes at chromosome 19q13. These genes include B cell lymphoma (Bcl)-3, which encodes elements of IκB from the NF-κB pathway [15–17]. Releases of the NF-κB proteins for intranuclear transfer allow them to bind to their target gene promoters that encode chemokines and cytokines, including persistently activated STATs [13, 16, 17].

Valproic acid (VPA) is commonly used to treat epilepsy. VPA exerts its therapeutic benefits through multiple mechanisms, including enhancement of GABAergic activity, depolarization induced by *N*-methyl-d-aspartic acid (NMDA) receptors, and/or inhibition of calcium channels and voltage-gated sodium [18–21]. Recent studies found that VPA is a class 1/II histone deacetylase inhibitor (HDCAi), where it inhibits histone deacetylase (HDCA) functions [18, 19]. Acetylated histone proteins exert their neuroprotective effects by reducing inflammation and inhibiting neuronal death [22, 23], which improve neurological functions in many neurological diseases, including cerebral ischemia [24], traumatic brain injury [25], and spinal cord injury [21, 26]. The role that VPA plays in SCI, as well as if VPA treatment could inhibit the activation of microglia and the subsequent inflammatory response after TBI, has not been studied or established.

The non-histone-binding protein complexes, such as the high-mobility group (HMG) family, the NF-kB, and the signal transducers and activators of transcription (STATs), are modified by post-translational modifications [14, 27]. Recent studies have confirmed that the transcriptional activities of NF-kB and STATs are closely related to their lysine acetylations, which are regulated by the balance between histone acetyltransferases (HATs) and histone deacetylases (HDACs) [28–30]. HDCAi seems to inhibit NF-kB transcriptional activity by maintaining the NF-kB acetylated (inactive) state and repressing the inflammatory response. Studies from Leus et al. [31] found that the expression levels of NF-kB are related to HDAC enzymatic activity and the levels of histone acetylation. These findings suggest that HDAC expression may be associated with NF-kB-mediated inflammation. The activation of STATs could alleviate a multitude of NF-kB-driven inflammatory and metabolic disorders [14, 27, 32]. There is no literature regarding the STAT-mediated nuclear translocation of NF-kB p65 subunit after a traumatic SCI. In the present study, we investigated if VPA, a class 1/II histone deacetylase inhibitor, attenuates the microglia-mediated neuroinflammatory response involving the interactive roles of HDAC, STATs, and NF-kB following a traumatic SCI.

## Methods

### Animals

Adult male Wistar rats (weighing 230–260 g) were purchased from the Fujian Medical University Experimental Animal Center. The rats were maintained in a clean, temperature-controlled environment (23 ± 2 °C) on a 12:12-h light/dark cycle with free access to food and water. The experimental protocols were in accordance with the guidelines for the care and use of laboratory animals by the Fujian Medical University Experimental Animal Ethics Committee (Fuzhou, China).

### Animal model and drug delivery

A total of 192 rats were randomly divided into the sham, the sham + VPA, the SCI, and the SCI + VPA group. Each of these groups had four subgroups (1-, 3-, 7-, and 14-day time points) (*n* = 12 per group). Six rats in each group were euthanized for the molecular biological and the biochemical experiments. The remaining rats in each group (*n* = 6) were used for evaluating the neurological function and the histological studies. The traumatic SCI rat model was established in accordance with the previous literature [33], under sodium pentobarbital anesthesia (50 mg/kg via intraperitoneal injection). An incision was made along the middle of the back, which exposed the paravertebral muscles and the vertebral laminas. A laminectomy was performed at the vertebral T9–T10 levels, exposing the dorsal surface of the cord without disrupting the dura. The cord was subjected to weight-drop impact to moderate contusion injury (10 g × 25 mm) using a 10-g metal rod at level T10. The Sham control rat group was subjected to a T9–T10 laminectomy without the weight-drop injury. Following the operation, manual bladder emptying was performed twice daily until the rats were able to urinate by themselves. Approximately 0.5 h after TBI, the rats in the sham + VPA and the SCI + VPA groups were administered VPA by intraperitoneal injection (300 mg/kg/day, diluted in dimethylsulfoxide; Sigma Aldrich, St Louis, MO, USA) once per day for three consecutive days [21, 34]. Forty microliters per kilogram fludarabine (flu) (5 mmol/L, diluted in dimethylsulfoxide) was administrated into the left lateral ventricle 24 h after intraperitoneal VPA to inhibit STAT1 signaling, which clarified the role of STAT1 in VPA neuroprotection [35]. The remainder of the groups was injected with the same dose of a dimethylsulfoxide, as a control.

### Behavioral assessment

The locomotor recovery was based on the Basso-Beattie-Bresnahan locomotion scale and the inclined plane test, in accordance with the previous reports [1, 2]. The locomotor recovery was evaluated at 1, 3, 7, and 14 days following the SCI. The Basso-Beattie-Bresnahan (BBB) test was graded on a scale of 0–21. A total score of 0 point indicated severe neurological deficits, and a score of 21 indicated normal performance. The inclined plane test was performed on a testing apparatus. The maximum angle where the rat retained its position for more than 5 s without falling was recorded. Behavioral assessments were performed at different time points by experimenters that were blinded to the group information.

### Measurement of blood-spinal cord barrier permeability

The integrity of the blood-spinal cord barrier was established by measuring the extravasation of Evans blue (Sigma Aldrich) [2]. The Evans blue dye (2% in saline; 4 mL/kg) was injected intravenously 2 h prior to the euthanasia at 1, 3, 7, and 14 days following the SCI. The mice were euthanized and then transcardially perfused with PBS, followed by an additional PBS containing 4% paraformaldehyde. Each tissue sample was immediately weighed and homogenized in a 1-mL 50% trichloroacetic acid solution. The samples were then centrifuged. The absorption of the supernatant was measured by a spectrophotometer (UV-1800 ENG 240V; Shimadzu Corporation, Japan) at a wavelength of 620 nm. The quantity of Evans blue was calculated with a standard curve and expressed as microgram of Evans blue per gram of brain tissue, using a standardized curve.

### Nissl staining

The spinal cord specimens near the lesion epicenter were fixed with formaldehyde. The formaldehyde-fixed specimens were embedded in paraffin and cut into 4-μm-thick sections. The sections were deparaffinized with xylene and rehydrated in a graded series of alcohol. Samples were treated with Nissl staining solution (Boster Biotech, Wuhan, China) for 5 min, and then mounted with neutral balsam. The apoptotic neurons were shrunken or contained vacuoles. The normal neurons had a relatively large and full soma, with round, large nuclei. Five areas were randomly selected to be examined with an inverted microscope (Leica, Wetzlar, Germany) by investigators who were blinded to the experimental groups.

### Immunohistochemical analysis

Formaldehyde-fixed specimens near the lesion epicenter were embedded in paraffin and cut into 4-μm-thick sections. These sections were deparaffinized with xylene and rehydrated in a graded series of alcohol. Antigen retrieval was performed by microwaving the samples in a citric acid buffer. The sections were incubated with a STAT1 (1:100; Cell Signaling Technology, Danvers, MA, USA) antibody, washed, and incubated with secondary antibody for 1 h at room temperature. These sections were mounted with neutral balsam. The negative control was prepared without the addition of the anti-STAT1 antibody. A total of five sections from each animal were used for quantification, and the signal intensity was evaluated as follows [36]: 0, no positive cells; 1, very few positive cells; 2, moderate number of positive cells; 3, many positive cells; and 4, the highest number of positive cells.

### Immunofluorescence analysis

Formaldehyde-fixed specimens were embedded in paraffin and cut into 4-μm-thick sections. The sections were deparaffinized with xylene and rehydrated in a graded series of alcohol, followed by antigen retrieval. The sections were incubated overnight at 4 °C with CD16 (1:200, Abcam, Cambridge, UK), CD206 (1:200; Abcam), neuronal nuclei (1:100; Boster Biotech, Wuhan, China), ionized calcium-binding adapter molecule (Iba)-1 (1:200; Santa Cruz Biotechnology, Santa Cruz, CA, USA), and GFAP (1:200; Abcam) antibodies. The sections were washed and incubated with secondary antibodies for 1 h at room temperature. The cell nuclei were stained with 4′,6-diamidino-2-phenylindole. The sections were then mounted with glycerol jelly mounting medium. The immunopositive cells from five randomly selected fields were counted under the inverted microscope (Leica, Wetzlar, Germany) at × 400 magnification by experimenters that were blinded to the experimental group.

### Terminal deoxynucleotidyl transferase dUTP nick-end labeling assay

Apoptotic cells were detected with a terminal deoxynucleotidyl transferase dUTP nick-end labeling (TUNEL) kit (Roche Diagnostics, Indianapolis, IN, USA) in accordance with the manufacturer’s instructions. Indicators of apoptosis included shrunken cell body, irregular shape, nuclear condensation, and brown diaminobenzidine staining, as observed by the inverted microscope (Leica, Wetzlar, Germany) at × 400 magnification. Average positive cell counts were calculated from the same sections in six rats per group with Image Pro Plus 7.0 by investigators who were blinded to the experimental groups.

### Enzyme-linked immunosorbent assay

Inflammatory factors in the brain tissue were detected with ELISA kits for TNF-α, IL-1β, IL-6, and IFN-γ (all from Boster Biotech). The Multiplex Microplate Reader (Molecular Devices, SpectraMax M5) was used to measure the absorbance value of ELISAs. The measured OD values were converted into a concentration value.

### Nuclear and cytoplasmic proteins extraction

The tissue samples were subjected to subcellular fractionation using the cytoplasmic and nuclear protein extraction kit (KeyGEN Biotech, KGP150), using hypotonic lysis buffer (20 mM HEPES (pH 7.4), 2 mM EGTA, 2 mM MgCl2) to extract the cytosolic protein, and using hypertonic lysis buffer (20 mM Tris/HCl, pH 7.6, 100 mM NaCl, 20 mM KCl, 1.5 mM MgCl2, 0.5% Nonidet P-40, and protease inhibitors) to extract the nuclear protein. The protein of the lysates was determined separately via Western blot by stripping the PVDF membranes and re-probed with LaminB1 (Cell Signaling Technology) as the nuclear control protein and β-actin (Boster Biotech) antibodies as the cytosolic control.

### Western blotting

Proteins were extracted with a radioimmunoprecipitation assay lysis buffer (sc-24948; Santa Cruz Biotechnology). The BCA method (KeyGEN Biotech, KGPBCA) was used for the protein quantitation. A total of 25 μg protein was separated by sodium dodecyl sulfate-polyacrylamide gel electrophoresis and transferred to a nitrocellulose membrane by wet transfer. The membrane was then blocked in 10% skim milk at room temperature for 2 h and then incubated at 4 °C overnight with primary antibodies against the following proteins: B cell lymphoma (Bcl)-2 (1:400), Bcl-2-associated X factor (Bax) (1:200), CD16 (1:200), and CD206 (1:200) (all from Abcam), and cleaved caspase-3 (1:200), Iba-1 (1:100), and NF-κB p65 (1:200) primary antibodies (all from Cell Signaling Technology), followed by incubation with the appropriate secondary antibodies (goat anti-rabbit IgG- HRP or goat anti-mouse IgG-HRP, 1:3500) at room temperature for 1 h. Immunoreactivity was visualized with the ECL Western Blotting Detection System (Millipore, Billerica, MA, USA). A gray value analysis was conducted with the UN-Scan-It 61 software (Silk Scientific Inc., Orem, UT, USA). Expression levels were normalized against β-actin (1:5000, Boster Biotech) or Lamin B1 (1:3000, Cell Signaling Technology).

### Co-immunoprecipitation

The spinal cord specimens near the lesion epicenter were incubated for 2 h at 4 °C with either 1 μg of STAT1 (Cell Signaling Technology) or 1 μg NF-κB p65 anti-acetylated lysine antibody (Cell Signaling Technology). A 10-μl volume of protein A/G agarose beads (Roche, Mannheim, Germany) was added to the sample, followed by overnight incubation. The agarose beads were washed three times with a lysis buffer after immunoprecipitation and centrifugation. The degree of acetylation of the STAT1 or the NF-κB p65 was analyzed with Western blotting, using an anti-acetylated lysine antibody (Cell Signaling Technology).

### HDAC activity assay and NF-κB DNA-binding activity assay

Nucleoproteins were extracted, where their concentrations were determined by bicinchoninic acid assay. A colorimetric HDAC fluorometric assay kit (BioVision, Mountain View, CA, USA) was used to detect the HDAC activity by measuring the absorbance at 405 nm on a microplate reader. A transcription factor binding assay colorimetric ELISA kit (Cayman Chemical, Ann Arbor, MI) was used to detect NF-κB p65 DNA-binding activity by measuring the absorbance at 450 nm on a microplate reader.

### Statistical analysis

The data was analyzed with SPSS v 19.0 software (SPSS Inc., Chicago, IL, USA). The results are expressed as the mean ± standard deviation. Comparisons between groups were made with the unpaired Student’s *t* test. Multiple group comparisons were assessed with one-way ANOVA. Post hoc multiple comparisons were performed using Student-Newman-Keuls tests. *P* < 0.05 was considered statistically significant.

## Results

### Neuroprotective effects of valproic acid on SCI

**Fig. 1:**
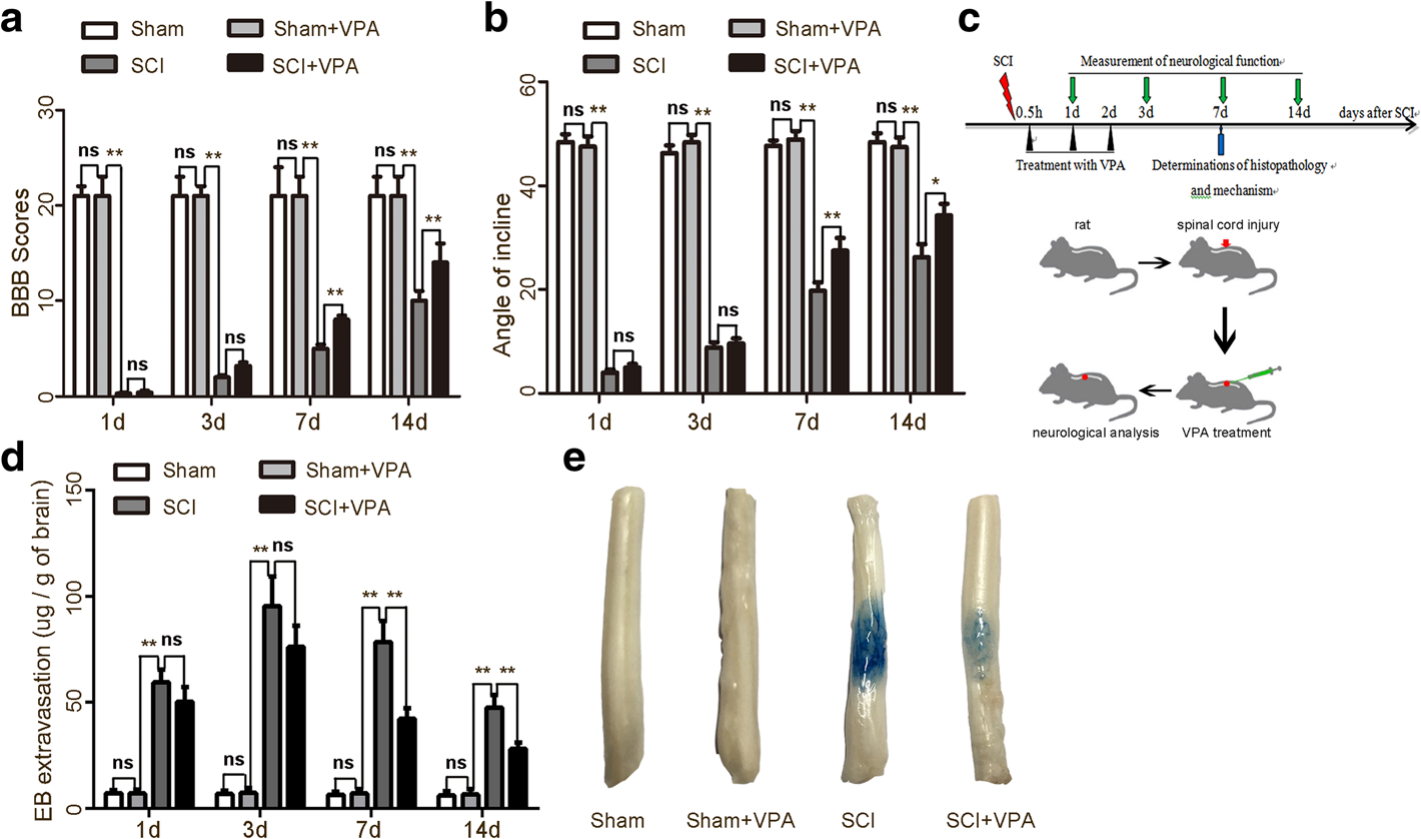
Neuroprotective effects of valproic acid on SCI. **a** Animals in the sham and sham + VPA groups obtained similar scores at the corresponding time points (score = 21). The neurological functions were severely impaired immediately after the SCI (*P* < 0.05). The BBB scores of these animals gradually returned to the control values. The most significant improvements were observed in the rats in the SCI + VPA group, 7 days after the SCI (8.17 ± 0.45 vs 5.03 ± 0.39) (*P* < 0.05). **b** The maximum angles were higher in SCI + VPA group than in the SCI group (27.44 ± 2.48 vs 19.75 ± 1.62) (*P* < 0.05). **c** Experimental scheme of VPA treatment after SCI. VPA was administered via intraperitoneal injection immediately following the SCI at level T10. **d**, **e** The SCI group obtained more Evans blue dye extravasation from 7 days after the SCI than the sham and the sham + VPA groups (*P* < 0.05). The SCI + VPA group had significantly less extravasation of Evans blue dye (*P* < 0.05) than the SCI group. Representative photos of the Evans blue dye extravasation in the experimental groups 7 days after the SCI. Values were expressed as mean ± standard deviation (*n* = 6 per group). N.S., *P* > 0.05, *P < 0.05, **P < 0.01

The BBB scores and the inclined plane test results were assessed to evaluate the effect of locomotion recovery following a SCI [1]. The BBB scores of the sham and the sham + VPA groups were unaltered after the operation. The rats in the sham and the sham + VPA groups obtained similar scores at the corresponding time points (scored 21). However, the neurological function was severely impaired immediately after the SCI (*P* < 0.05). The BBB scores of these animals gradually returned to the control values, with significant improvement observed in the rats of the SCI + VPA group, 7 days after the SCI (8.17 ± 0.45 vs 5.03 ± 0.39) (*P* < 0.05) (Fig. 1a). The results of the inclined plane test also showed that the maximum angles were higher in SCI + VPA group than in SCI group (27.44 ± 2.48 vs 19.75 ± 1.62) (*P* < 0.05) (Fig. 1b).

The blood-spinal cord barrier (BSCB) permeability was investigated by measuring the extravasation of the Evans blue [2]. The results demonstrated that the SCI group obtained more Evans blue dye extravasation 7 days after the SCI than the sham and the sham + VPA groups (*P* < 0.05). The SCI + VPA group obtained significantly less extravasation of Evans blue dye than the SCI group (*P* < 0.05) (Fig. 1c–e). The results of the behavioral assessment and BSCB permeability demonstrated that VPA treatment exerted a neuroprotective effect in the lesioned spinal cord 7 days after the SCI. So, 7 days after the SCI was chosen as the time point for the following study.

### VPA protects neurons against SCI-induced neuronal apoptosis

**Fig. 2:**
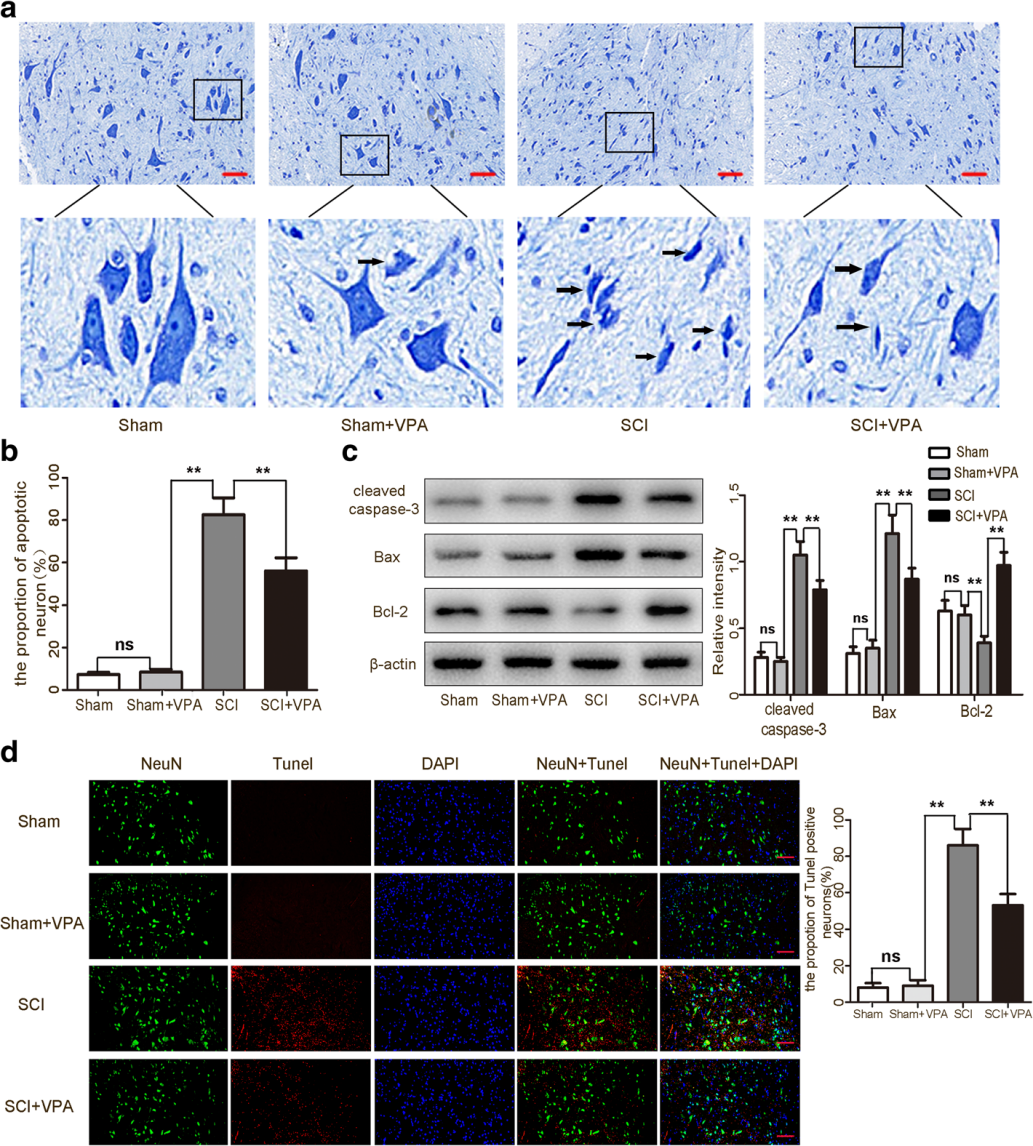
VPA protects neurons against SCI-induced neuronal apoptosis in the lesioned spinal cord 7 days after SCI. **a**, **b** The sham group and the sham + VPA group obtained a low apoptotic fraction of neurons 7 days after the SCI. The percentage of apoptotic cells was higher in the SCI than in the sham group (*P* < 0.05). The apoptotic fraction was significantly lower in the SCI + VPA than in the SCI group (*P* < 0.05). Representative photomicrographs of the Nissl-stained neurons are shown. Enlarged images of boxed areas are shown below. The arrows indicate the apoptotic neurons. **c** Western blot analyses revealed that the SCI resulted in the upregulation of apoptotic factors in the lesioned spinal cord 7 days after the SCI. The cleaved caspase-3 and Bax levels decreased and the anti-apoptotic factor Bcl-2 increased in the SCI + VPA group more than the SCI group (*P* < 0.05). **d** The TUNEL staining demonstrated that the TUNEL-positive neurons decreased significantly more in the VPA + SCI group than in the SCI group. Representative photomicrographs of the TUNEL-positive neurons are shown. The arrows indicate the apoptotic neurons. Values were expressed as mean ± standard deviation (*n* = 6 per group). N.S., *P* > 0.05, *P < 0.05, **P < 0.01. Scale bars = 50 μm

Nissl staining was used to identify the apoptotic neurons in the lesioned spinal cord [1]. The rates in the sham group and the sham + VPA group obtained a low apoptotic fraction of neurons 7 days after the SCI. The percentage of apoptotic cells was higher in the SCI group than in the sham group (*P* < 0.05). The apoptotic fraction was significantly lower in the SCI + VPA group than in the SCI group (*P* < 0.05) (Fig. 2a,b). Western blot analyses revealed that the SCI resulted in the upregulation of apoptotic factors in the lesioned spinal cord 7 days after the SCI. The cleaved caspase-3 and Bax levels decreased in the SCI + VPA group more than the SCI group. The anti-apoptotic factor Bcl-2 increased in the SCI + VPA group (*P* < 0.05) (Fig. 2c). The TUNEL assay confirmed that the percentage of TUNEL-positive neurons was lower in the VPA + SCI group than in the SCI group (Fig. 2d). These results suggested that the VPA treatment had no obvious effect on the normal spinal cord but did exert a neuroprotective effect in the lesioned spinal cord.

### VPA promotes microglia polarization toward M2 and alleviates microglia-mediated inflammatory response

**Fig. 3:**
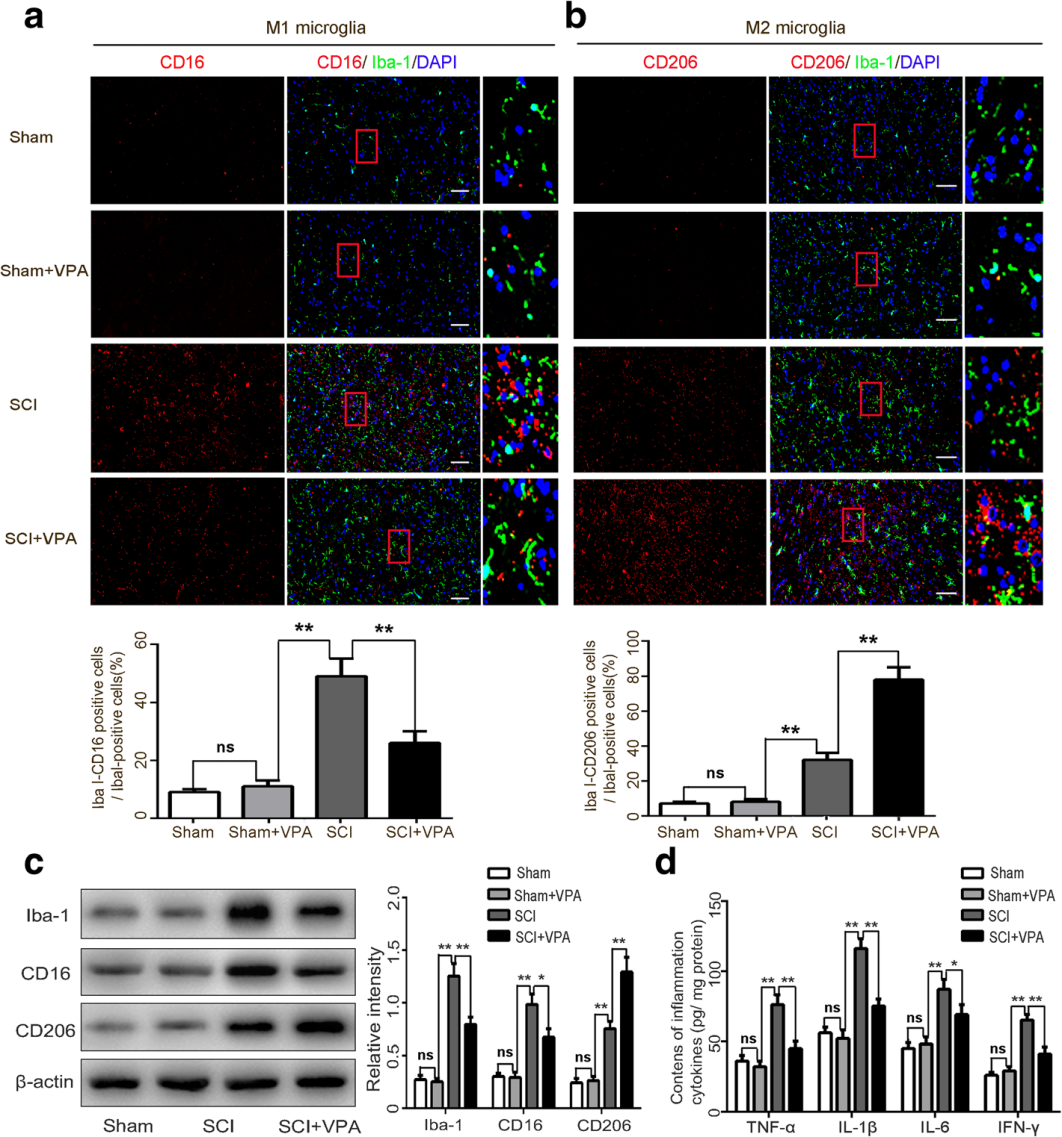
VPA promotes microglia polarization toward M2 and alleviates microglia-mediated inflammatory response. **a**, **b** The double immunohistochemical staining for the microglia (Iba1+) and the M1-associated marker (CD16+) or the M2-associated marker (CD206+) 7 days after the SCI was assessed. The microglia labeled with the M1 (CD16+) increased after the SCI but significantly decreased following the VPA treatment. The M2 (CD206+) increased following the VPA treatment. Representative photomicrographs of the CD16 or the CD206-positive microglia are shown. Enlarged images of boxed areas are shown on the right. **c** Western blot analysis showed that the M1 phenotype microglial proteins (CD16 and Iba-1) were significantly inhibited, whereas the M2 phenotype microglial protein (CD206) increased following the VPA treatment. **d** The VPA treatment significantly decreased the SCI-induced enhancements of TNF-α, IL-1β, IL-6, and IFN-γ (*P* < 0.05). Values were expressed as mean ± standard deviation (*n* = 6 per group). N.S., *P* > 0.05, *P < 0.05, **P < 0.01. Scale bars = 50 μm

A phenotypic transition of the microglia from the anti-inflammatory (M2)-like to the pro-inflammatory (M1)-like played a crucial role in the microglial activation and its mediated neuroinflammatory response [4, 37]. The microglia polarization shift was tested with double immunohistochemical staining on the microglia (Iba-1) and the M1-associated marker (CD16) or the M2-associated marker (CD206) 7 days after the SCI was assessed. The microglia labeled with the M1 (CD16+) increased after the SCI but significantly decreased after the VPA treatment. The M2 (CD206+) increased further after the VPA treatment. The VPA encouraged the phenotypic shift of the microglia from M1 to M2 (Fig. 3a,b). The Western blot analysis showed that the M1 phenotype microglial proteins (CD16 and Iba-1) were significantly inhibited, whereas the M2 phenotype microglial proteins (CD206) increased after the VPA treatment (Fig. 3c). The expression levels of the inflammatory factors (TNF-α, IL-1β, IL-6, and IFN-γ) were measured after the SCI with an ELISA kit. The results showed that the SCI group had significantly higher expression levels of inflammatory factors than the sham and the sham + VPA groups, while the VPA treatment decreased the SCI-induced enhancement of these factors (*P* < 0.05) (Fig. 3d). These findings suggested that the VPA treatment shifted the microglia polarization toward the M2 phenotype and alleviated the microglia-mediated inflammatory response.

### VPA inhibits HDAC3 expression in the lesioned spinal cord

**Fig. 4:**
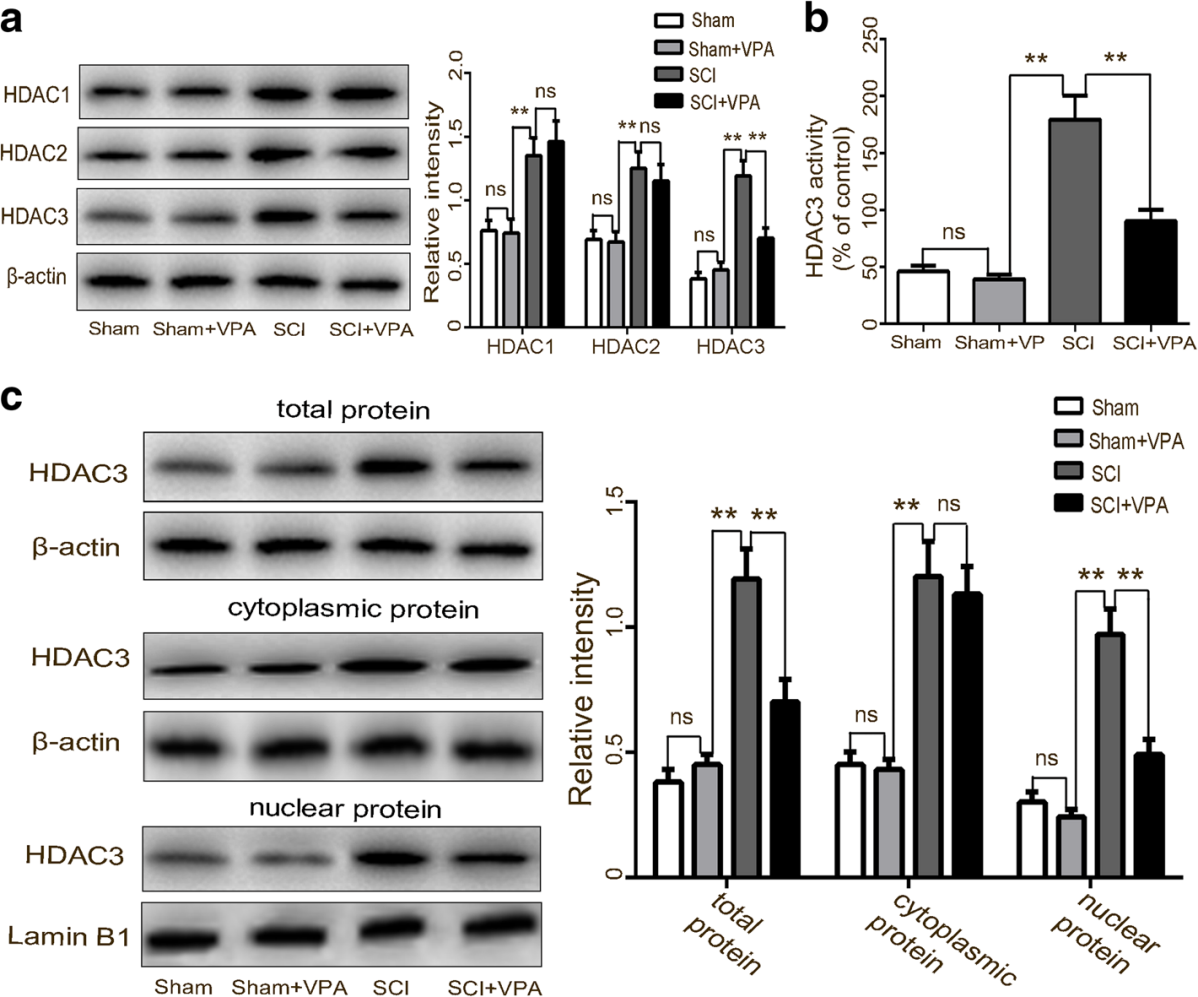
VPA inhibits HDAC3 expression in the lesioned spinal cord. **a** Western blot analysis demonstrated that the expressions of HDAC 1–3 were expressed at a low level in the sham group, but were upregulated in the lesioned spinal cord 7 days after the SCI. The VPA treatment reduced the HDAC3 protein expression more than the SCI group (*P* < 0.05), without affecting the HDAC1 and the HDAC2 protein expressions (*P* > 0.05). **b** The HDAC3 activity in the SCI + VPA group was significantly less than in the SCI group (*P* < 0.05). **c** The expressions of HDAC3 in the cytosol, nuclei, and in total protein levels of the cells from the lesioned cortices increased after the SCI. The VPA treatment effectively decreased the HDAC3 expression in the nuclear and in the total protein of cells from the lesioned spinal cord (*P* < 0.05) but not in the cytosol protein (*P* > 0.05). Values were expressed as mean ± standard deviation (*n* = 6 per group). N.S., *P* > 0.05, *P < 0.05, **P < 0.01

**Fig. 5:**
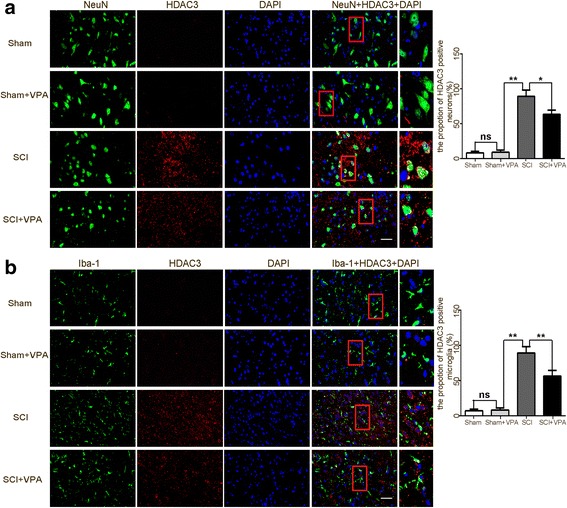
HDAC3 expression was inhibited in both neurons and microglia after VPA treatment. Double immunohistochemical staining was used to assess the neuron (NeuN+) and the microglia (Iba-1+) in the lesioned spinal cord 7 days after the SCI. **a** The SCI enhanced the expression of the HDAC3 in neurons (NeuN+), which significantly decreased after the VPA treatment. Representative photomicrographs of the HDAC3-positive neurons are shown. Enlarged images of boxed areas are shown on the right. **b** The SCI enhanced the expression of HDAC3 in microglia (Iba-1+), which was significantly decreased following the VPA treatment. Representative photomicrographs of HDAC3-positive microglia are shown. Enlarged images of boxed areas are shown on the right. Scale bars = 50 μm

There have been several roles established for HDAC 1–3, where it plays key roles in pro-inflammatory gene expression in inflammatory diseases [14, 38]. The results showed that the HDAC 1–3 was expressed at a low level in the sham group but were upregulated in the lesioned spinal cord 7 days after the SCI. The treatment with the HDAC inhibitor (VPA) reduced HDAC3 protein expression (*P* < 0.05), without affecting the HDAC1 and the HDAC2 protein expressions (*P* > 0.05) (Fig. 4a). The increase of HDAC3 activity induced by the SCI was mitigated by the VPA (Fig. 4b). The Western blot analysis demonstrated that the expressions of HDAC3 in the cytosol, the nuclei, and in the total protein levels of the cells from the lesioned cortices increased after the SCI. The VPA treatment effectively decreased the HDAC3 expression in the nuclear and in the total protein of cells from these lesioned cortices (*P* < 0.05) but not in the cytosol protein (*P* > 0.05) (Fig. 4c). The double immunofluorescent staining was used to assess the HDAC3 expressions in the neuron (NeuN +) and microglia (Iba-1 +) of the lesioned spinal cord 7 days after the SCI. The expression levels of HDAC3 were higher in the SCI group than in the sham and the sham + VPA groups. The HDAC3 expression was inhibited in both neurons and microglia following the VPA treatment (Fig. 5a,b). These results indicated that VPA exerted neuroprotective effects and inhibited microglia-mediated inflammatory response after SCI, perhaps dependent on HDAC3 expression and activity.

### Effect of VPA on STAT1 acetylation and NF-κB signaling pathway

**Fig. 6:**
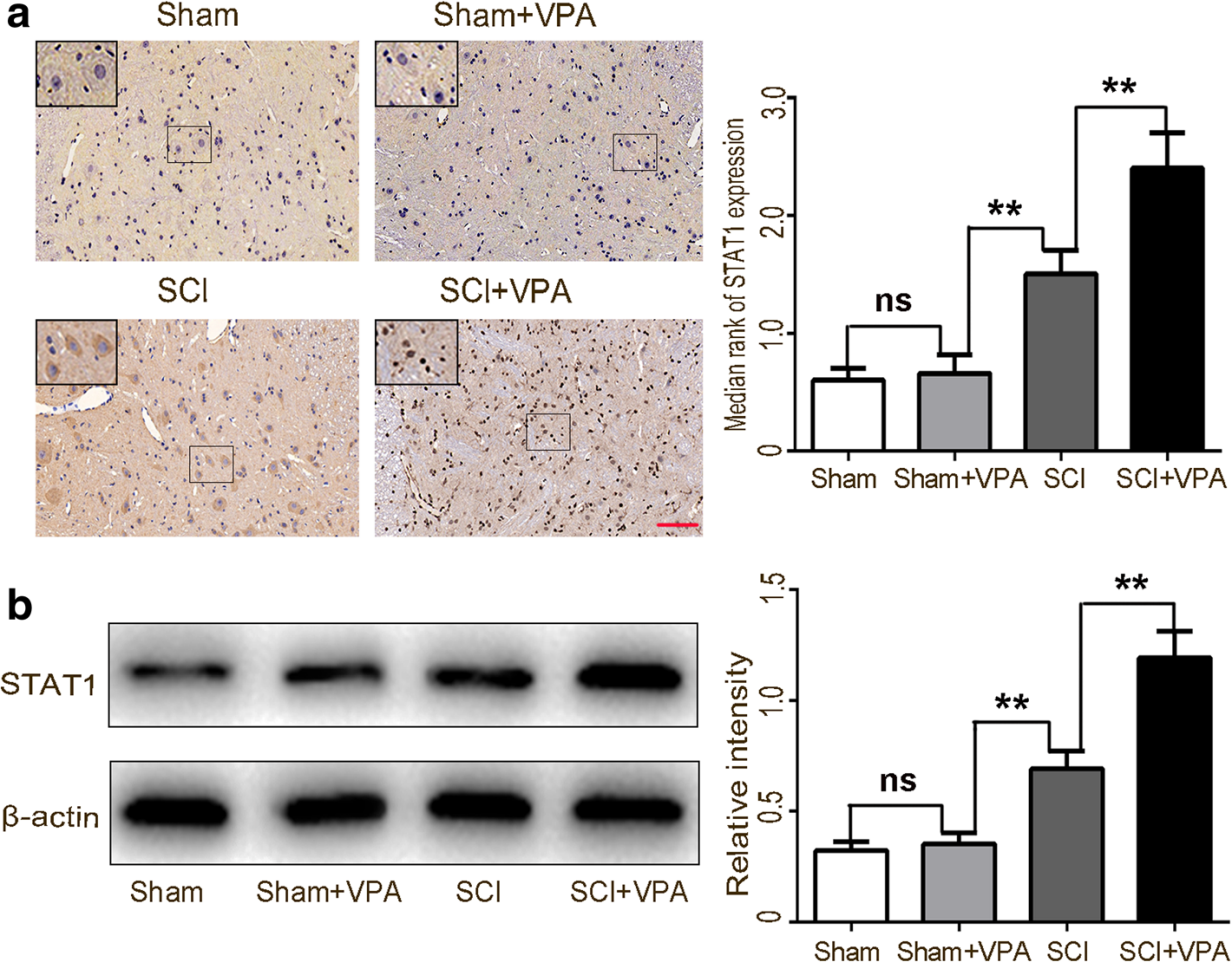
VPA treatment elevates STAT1 expression in the lesioned spinal cord 7 days after SCI. **a** STAT1 expressions were higher in the SCI than in the sham group. The STAT1 protein levels were upregulated following the VPA treatment (*P* < 0.05). Enlarged images of boxed areas are shown on the upper left. **b** Western blot analysis showed that the STAT1 protein levels were upregulated after the VPA treatment (*P* < 0.05). Values were expressed as mean ± standard deviation (*n* = 6 per group). N.S., *P* > 0.05, *P < 0.05, **P < 0.01. Scale bars = 50 μm

NF-κB is considered the central transcription factor of inflammatory mediators, where it plays a vital role in microglial activation [8, 9]. The activation of STATs had potential in alleviating a multitude of NF-kB-driven inflammatory and metabolic disorders [14, 27]. The STAT1 expression was evaluated by immunohistochemistry and Western blotting in the lesioned spinal cord 7 days following the SCI. The STAT1 expressions were higher in the SCI group than in the sham group (*P* < 0.05). The STAT1 protein levels were upregulated after the VPA treatment (Fig. 6a,b). Our studies have shown that the VPA suppressed HDAC3 expression and activity. We speculated that this would lead to an increased STAT1 expression via downregulation of the HDAC3 expression.

**Fig. 7:**
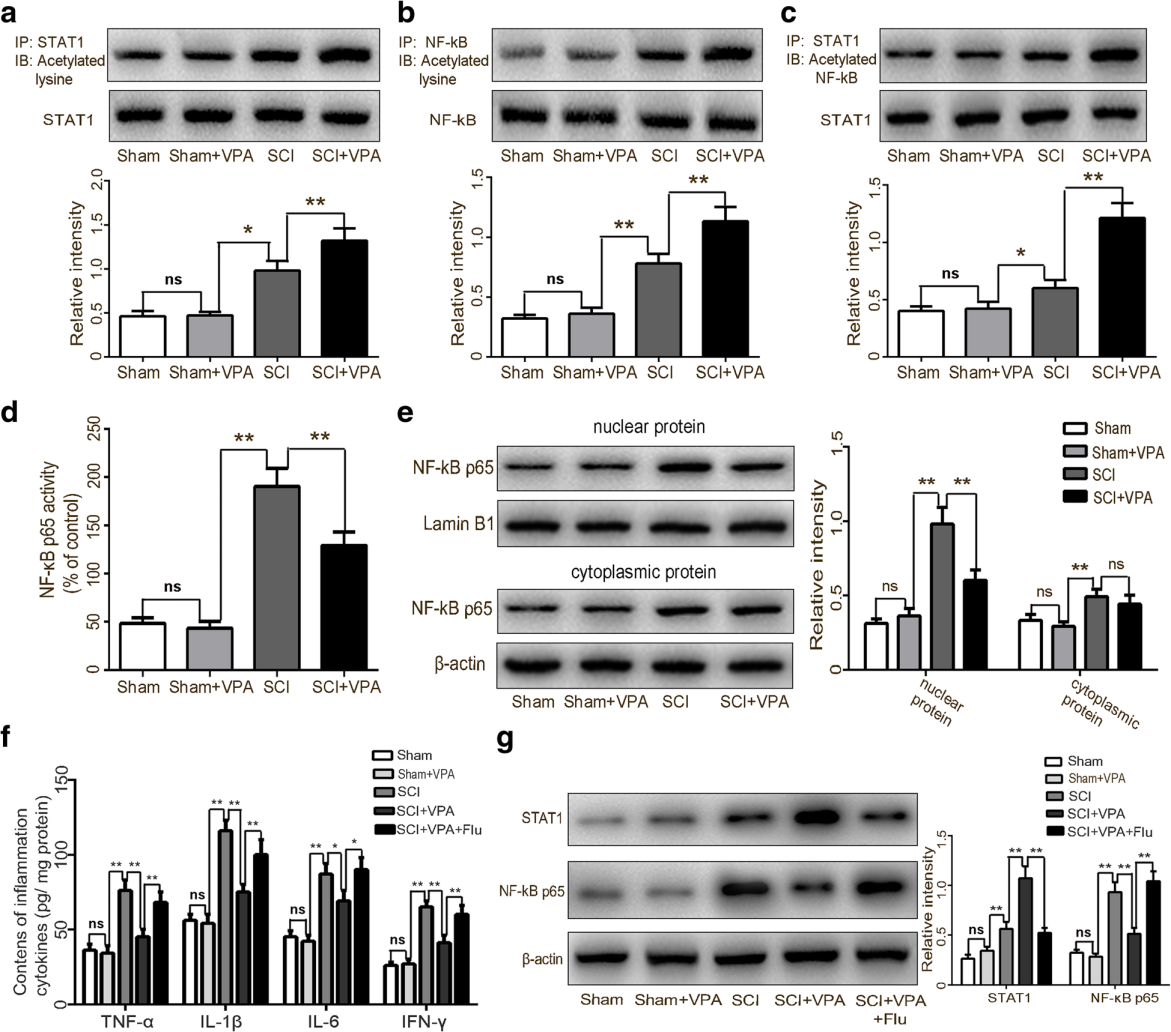
VPA treatment suppressed the NF-κB pathway via elevating STAT1 expression in the lesioned spinal cord 7 days after SCI. **a** Co-IP analysis showed a greater elevation in the STAT1 after the VPA treatment than in the SCI group (*P* < 0.05). **b** Co-IP analysis showed a greater elevation in the NF-κB acetylation after the VPA treatment than in the SCI group (*P* < 0.05). **c** The acetylated STAT1 formed a complex with the nuclear NF-κB p65. The VPA induced significant interactions between STAT1 and NF-κB p65 after the SCI (*P* < 0.05). **d** Significantly, less NF-κB p65 DNA-binding activity was observed in the SCI + VPA group than in the SCI group. **e** The VPA treatment inhibited NF-κB p65 nuclear translocation and expression (*P* < 0.05). **f** The inhibitory effect of the VPA treatment on the neuroinflammatory response was reversed by the pharmacological inhibition of STAT1 (fludarabine, flu). **g** The inhibitory effect of the VPA treatment on the NF-κB pathway was reversed by pharmacological inhibition of STAT1. Values were expressed as mean ± standard deviation (*n* = 6 per group). N.S., *P* > 0.05, *P < 0.05, **P < 0.01

Lysine acetylation of the STAT1 had been shown to be dispensable for the NF-kB pathway activity in response to damaged stimuli [14]. We investigated the change in the NF-κB signaling following the VPA treatment in order to reconcile the contradictions between the VPA-induced expression of the STAT1 and the inhibition of NF-kB pathway activity by the VPA treatment after a SCI. The co-IP assay confirmed that the VPA increased the STAT1 acetylation (Fig. 7a) and the NF-κB p65 acetylation (Fig. 7b). The acetylated STAT1 formed a complex with the nuclear NF-κB p65. The VPA induced significant interactions between STAT1 and NF-κB p65 after the SCI (Fig. 7c). A transcription factor binding assay colorimetric ELISA kit was used to measure the NF-κB p65 DNA-binding activity. A significant reduction in the NF-κB p65 DNA-binding activity was observed in the SCI + VPA group when compared to the SCI group (Fig. 7d). The VPA treatment inhibited the NF-κB p65 nuclear translocation and the expression. The VPA treatment induced significant interactions between the STAT1 and the NF-κB p65, thereby inhibiting its nuclear translocation and its DNA-binding activity following a SCI (Fig. 7e). The inhibitory effects of the VPA treatment on the inflammatory response and the STAT1/NF-κB axis activation were reversed by the pharmacological inhibition of STAT1, which suggested that the anti-inflammation effect of the VPA was reliant on the STAT1 expression (Fig. 7f,g).

## Discussion

Accumulating evidence have demonstrated the benefits of VPA against SCI-induced neural damage and secondary pathological processes [18, 21]. The present study also supports the view that VPA is a suitable therapeutic candidate against SCI-induced neurological deficits. VPA treatment reduced brain edema and BBB permeability and improved neurological function after SCI. Furthermore, VPA treatment protected neurons against SCI-induced neuronal apoptosis by inhibiting the expression of the pro-apoptotic factors, cleaved caspase-3 and Bax, and increasing expression of the anti-apoptotic factor, Bcl-2. The VPA works as a histone deacetylase inhibitor to directly inhibit HDACs, which have been implicated in many biological activities [21, 38, 39]. The present study confirmed that the HDAC3 expression was upregulated in the lesioned spinal cord, suggesting that the histone deacetylation played an important role in post-traumatic secondary spinal cord injury. The VPA treatment reduced the nuclear HDAC3 expression and inhibited its activity following the SCI, thereby suppressing neuronal apoptosis. These findings indicated that the VPA exerted neuroprotective effects by inhibiting HDAC3 expression and activity.

The microglia-mediated inflammatory response plays an important role in secondary spinal cord injury after a SCI [4, 40, 41]. A phenotypic transition of microglia from the anti-inflammatory (M2)-like to the pro-inflammatory (M1)-like play a crucial role in the microglial activation and its mediation of neuroinflammatory response [4, 40, 41]. Our study showed that the levels of activated microglia and the inflammatory cytokines (TNF-α, IL-1β, IL-6, and IFN-γ) in the spinal cord tissue increased after the SCI, which were linked to the BSCB permeability and the neurological function scores. The inhibition of HDAC activity following the VPA treatment promoted the phenotypic shift of microglia from the M1 to the M2 phenotype, as well as inhibiting microglial activation and reducing the expressions of inflammatory cytokines in vivo. The NF-κB is considered to be the central transcription factor of inflammatory mediators, where it plays a crucial role in inflammation [8, 9]. The VPA treatment significantly weakened the NF-κB p65 nuclear translocation and its transcriptional activity following the SCI, which showed that the VPA exerted neuroprotective effects.

The non-histone-binding protein complexes, including the HMG family, the NF-kB, and the STATs, are modified by post-translational modifications [27]. The NF-κB signaling pathway is activated by post-translational modification. The phosphorylation and the methylation on serine residues within two nuclear localization signals (NLS) of the NF-κB in monocytes/macrophages accelerate its nuclear translocation, as well as its transcriptional activity [3, 14, 42]. The post-translational acetylation of NF-κB is also reported to promote its transcriptional activity, which is regulated by the balance between the HDACs and the histone acetyltransferases (HATs) [28–30]. The HDCAi seems to inhibit NF-kB transcriptional activity by maintaining the NF-kB acetylated (inactive) state, which represses the inflammatory response [18, 38]. The Co-IP analysis showed that the VPA increased the NF-kB p65 acetylation, which inhibited the NF-kB p65 nuclear translocation and its transcriptional activity. The HDCAi has been shown to alter the equilibrium between the HDACs and the HATs while inducing the STAT1 acetylation on the Lys 410 and the Lys 413 sites [14, 38]. The Co-IP analysis also showed that the presence of the STAT1 protein in acetyl-lysine immunoprecipitate fractions confirmed increased STAT1 acetylation following the VPA treatment. The VPA-mediated upregulation of the acetylation and the expression of STAT1 was likely to be due to the reduced HDAC3 translocation to the nucleus and the activity.

The STAT1 pathway plays a critical role in mediating the NF-kB p65 nuclear translocation [13, 14]. The lysine acetylation of the STAT1 seemed to be dispensable for the NF-kB releasing from its inhibitor IκB inhibition in response to damage stimuli [14]. The evidence regarding the cross-talk association of the STAT1 and the NF-kB pathways should be further investigated. The current study provided a novel counter-regulatory relationship between the attenuation of the SCI-mediated NF-kB p65 activity, which seemed to be regulated by the interactions of STAT1 and HDACs. The Co-IP assays indicated that the VPA induced significant interactions between the STAT1 and the NF-κB p65 after the SCI. The acetylated STAT1 formed a complex with the nuclear NF-κB p65, which inhibited its DNA-binding activity and weakened the central inflammatory response following the SCI. The inhibitory effects of the VPA treatment on the inflammatory response and on the STAT1/NF-κB axis activation were reversed by the pharmacological inhibition of STAT1, suggesting that the anti-inflammation effect of the VPA was dependent on STAT1 expression. Future studies involving STAT1 knockout mice should be performed to investigate the mechanisms involved in the VPA-mediated activation of STAT1 and the subsequent inhibition of the NF-κB pathway. Additional in vitro experiments are required to establish the direct effect of the VPA treatment on neuron and microglia activation.

## Conclusions

The VPA-mediated upregulation of the acetylation of the STAT1/NF-κB pathway was likely caused by the reduced HDAC3 translocation between the nucleus and the HDAC3 activity. The VPA inflammation inhibition affected the acetylation status of NF-kB p65 and the complex with the NF-κB p65 and the STAT1. This indicated that the VPA treatment suppressed the expression and the activity of HDAC3 and enhanced the STAT1 and the NF-κB p65 acetylation following the SCI. The acetylation status of the NF-kB p65 and the complex with NF-κB p65 and STAT1 inhibited the NF-kB p65 transcriptional activity and weakened the microglia-mediated central inflammatory response following the SCI.

## Abbreviations

BBB: Basso-Beattie-Bresnahan locomotion scale
BSCB: Blood-spinal cord barrier
Co-IP: Co-immunoprecipitation
ELISA: Enzyme-linked immunosorbent assay
flu: Fludarabine
HATs: Histone acetyltransferases
HDACs: Histone deacetylases
HMG: High-mobility group
IFN: Interferon
IL: Interleukin
NF-κB: Nuclear factor-κB
NMDA: *N*-methyl-d-aspartic acid
SCI: Spinal cord injury
STATs: Signal transducers and activators of transcriptions
TNF: Tumor necrosis factor
TUNEL: Terminal deoxynucleotidyl transferase dUTP nick-end labeling
VPA: Valproic acid
